# Modeling of active skeletal muscles: a 3D continuum approach incorporating multiple muscle interactions

**DOI:** 10.3389/fbioe.2023.1153692

**Published:** 2023-05-18

**Authors:** Wei Zeng, Donald R. Hume, Yongtao Lu, Clare K. Fitzpatrick, Colton Babcock, Casey A. Myers, Paul J. Rullkoetter, Kevin B. Shelburne

**Affiliations:** ^1^ Center for Orthopaedic Biomechanics, University of Denver, Denver, CO, United States; ^2^ Department of Mechanical Engineering, New York Institute of Technology, New York, NY, United States; ^3^ Department of Engineering Mechanics, Dalian University of Technology, Dalian, China; ^4^ Mechanical and Biomedical Engineering, Boise State University, Boise, ID, United States

**Keywords:** active skeletal muscle, Hill-type muscle model, finite element analysis, parametric study, quadriceps, muscle interactions

## Abstract

Skeletal muscles have a highly organized hierarchical structure, whose main function is to generate forces for movement and stability. To understand the complex heterogeneous behaviors of muscles, computational modeling has advanced as a non-invasive approach to evaluate relevant mechanical quantities. Aiming to improve musculoskeletal predictions, this paper presents a framework for modeling 3D deformable muscles that includes continuum constitutive representation, parametric determination, model validation, fiber distribution estimation, and integration of multiple muscles into a system level for joint motion simulation. The passive and active muscle properties were modeled based on the strain energy approach with Hill-type hyperelastic constitutive laws. A parametric study was conducted to validate the model using experimental datasets of passive and active rabbit leg muscles. The active muscle model with calibrated material parameters was then implemented to simulate knee bending during a squat with multiple quadriceps muscles. A computational fluid dynamics (CFD) fiber simulation approach was utilized to estimate the fiber arrangements for each muscle, and a cohesive contact approach was applied to simulate the interactions among muscles. The single muscle simulation results showed that both passive and active muscle elongation responses matched the range of the testing data. The dynamic simulation of knee flexion and extension showed the predictive capability of the model for estimating the active quadriceps responses, which indicates that the presented modeling pipeline is effective and stable for simulating multiple muscle configurations. This work provided an effective framework of a 3D continuum muscle model for complex muscle behavior simulation, which will facilitate additional computational and experimental studies of skeletal muscle mechanics. This study will offer valuable insight into the future development of multiscale neuromuscular models and applications of these models to a wide variety of relevant areas such as biomechanics and clinical research.

## 1 Introduction

As a major type of human muscle, skeletal muscle provides locomotion, maintains posture, and stabilizes bones and joints (Fung, 1993). A single skeletal muscle is an organ with a hierarchical structure, composed of complex fibrous actuators organized in different levels of aggregation (Gotti et al., 2020). Each skeletal muscle is wrapped in the epimysium (or deep fascia), an irregular fibrous connective tissue sheath which separates individual muscle and allows the enveloped muscle to move independently. As such, skeletal muscle behaviors are highly heterogeneous, and the muscle properties are extremely complicated (Blemker, 2017). Due to the structural, architectural, and mechanical complexity of the skeletal muscles, *in vivo* characterization of some physical quantities cannot be made without highly invasive methods (Dao and Tho, 2018). Computational modeling of skeletal muscles has become an indispensable modern method to determine these quantities, understand the complex heterogeneous behaviors of skeletal muscles, and the role of muscle pathology in movement biomechanics.

Phenomenological and biophysical approaches have been developed to model active muscle, including the most widely used Hill-type (Hill, 1938) and Huxley-type (Huxley, 1957) muscle models. The biophysical Huxley-type model for muscle contraction considered the cross-bridges and dynamics of myosin filaments within muscle. Usually, it needs a detailed set of experimental data to calibrate the model, and it is computationally expensive (Khodaei et al., 2013). Recent advances in multiscale biophysical muscle modeling have enabled simulation from the microscopic half-sarcomere level to the whole-muscle organ level (Röhrle et al., 2012; Heidlauf et al., 2016; Spyrou et al., 2019). As the most widely used landmark model to study mechanical behaviors of muscles, the Hill-type model (Hill, 1938) uses a macroscopic phenomenological approach to describe active muscle properties by introducing a contractile component with force–length and force–velocity relations for the entire muscle in one dimension (Zajac, 1989). To enhance the one-dimensional (1D) Hill-type model’s biofidelity and to represent the shape of muscles more realistically, it has been extended into three-dimensional (3D) Hill-type models for musculotendon dynamics. Most of the developed Hill-type phenomenological models for active muscles included passive and active components (Dao and Tho, 2018). The passive component has been commonly characterized by hyperelastic materials using the Neo-Hookean model (Göktepe et al., 2014), the Mooney-Rivlin models (Wu et al., 2014), or the Ogden model (Hedenstierna et al., 2008; Mo et al., 2019). The sophisticated Ogden model can accurately describe the nonlinear response of highly deformable tissues but is computationally expensive and requires more material parameters than the other two models. The Neo-Hookean type model, which is a popular choice, was adopted in the current study for its computational efficiency and ease of implementation but it can be limited in its applicability to tissues with highly deformable and compressible states. The definition of the active component of muscles requires the utilization of hierarchical fibers and their activation mechanism, which can be modeled by various approaches.

Over the past 20 years, there have been many exciting developments in constitutive formulations for active muscle behaviors. Based on the model of the cardiac muscle proposed by Humphrey and Yin (1987), Martins et al. (1998 and 2006) modeled skeletal muscles as a fiber-reinforced incompressible hyperelastic composite. Fernandez et al. (2005) presented a finite element (FE) framework for modelling the mechanical response of the rectus femoris muscle to a flexion loading. However, the simulations in that study simplified several aspects including the spatial distribution of material properties and excitation-contraction coupling. A number of studies have modeled the skeletal muscles by a combination of Hill-type 1D truss elements (or beam elements) for the active part and 3D solid elements for the passive part, i.e., discrete approaches for modeling skeletal muscles. For instance, Hedenstierna et al. (2008) modeled active muscles using a combination of passive non-linear, hyperelastic viscoelastic solid elements and active Hill-type truss elements, which were validated by rabbit leg muscles with simple geometry; Feller et al. (2016) incorporated a strain-dependent muscle activation scheme using 1D beam elements into the neck of a full human body model for a low-loading condition through a head fall test. Aiming to predict injury responses of lower limb-pelvis during the whole joint movement; Mo et al. (2018) developed a lower extremity model with active muscles in a recent study. The muscles were modeled as a 3D passive unit with an isotropic hyperelastic material and Hill-type 1D truss elements, which were created surrounding the 3D mesh of passive unit. They also implemented a muscle control strategy for combining multibody dynamics and finite element analysis (FEA) (Mo et al., 2019). In these studies, the distribution of the multiple truss or beam elements needs to be defined based on the mesh of solid elements which was a tedious process and can cause numerical instability issues.

There have been several advancements in the constitutive modeling for simulating complex active skeletal muscle behaviors at the macroscopic scale, including tissue strain, intramuscular pressure, muscle force, fatigue, and parameter identification (Blemker et al., 2005; Grasa et al., 2011; Khodaei et al., 2013; Lu et al., 2010 and 2011a; Tang et al., 2007 and 2009; Wheatley et al., 2018; Klotz et al., 2021). For example, Blemker et al. (2005) created a 3D FE model of the biceps brachii and compared the predicted tissue strains with experimental data. Lu et al. (2010) developed a transversely visco-hyperelastic model of skeletal muscle tissue under high strain rates. The model was validated using experimental studies of rabbit tibialis anterior (TA) muscle with simplified cylindrical geometry and a uniform muscle fiber direction. A similar study has been presented by Khodaei et al. (2013) using a transversely isotropic visco-hyperelastic model. Wheatley et al. (2018) presented an FE model of rabbit TA with a hyperporo-viscoelastic constitutive law to predict intramuscular pressure and muscle force under passive and active conditions.

In addition to the complexity of constitutive representations of skeletal muscles, the definition of fiber trajectories or architecture is a critical step to provide important input to the muscle model. There are several different approaches to characterize the arrangement of fibers, such as parallel-fibered architecture using parallel fiber distribution along a uniform direction or at some specific angle (Blemker et al., 2005; Clemen et al., 2017; Lu et al., 2010 and 2011a; Tang et al., 2007 and 2009), bipennate fiber orientation (Fernandez et al., 2005), and fusiform-shaped fiber distribution (Grasa et al., 2011). Other approaches include defining a fiber map template which is morphable to the target muscle geometry (Blemker and Delp, 2005; Röhrle and Pullan, 2007), using a method with non-uniform rational B-splines (NURBS) to characterize the fiber orientation (Lu et al., 2011b). However, these approaches have their own limitations. For instance, the standard fiber map templates may not be adequate to characterize various muscle architectures (Blemker, 2017), and alternative approaches either rely on oversimplified fiber orientations (Blemker et al., 2005; Tang et al., 2007 and 2009; Fernandez et al., 2005; Grasa et al., 2011) or encounter challenges with characterizing muscles that have multiple branches (Lu et al., 2011b). This study employed a physics-based method that utilizes computational fluid dynamics (CFD) to determine the muscle fiber architecture of muscles with realistic geometry. Inouye et al. (2015) recently proposed this approach and Handsfield et al. (2017) demonstrated it to be effective in predicting subject-specific muscle fiber patterns.

While many computational approaches have been developed to model the complex mechanical behaviors of individual active muscles, few studies have incorporated multiple muscles into a system-level model (Dao and Tho, 2018). For example, Elyasi et al. (2022) combined FE and multibody models into a lower limb model and to predict surgery’s impact on patellar position, but the model has limitations due to simplified anatomical details and material properties (e.g., all muscles were modelled as a passive state). Similarly, Spyrou and Aravas (2012) developed an FE model of the lower leg with active muscles to simulate plantar flexion but did not account for muscle interactions or detailed anatomical relationships. To date, very few studies have been able to model the complex anatomical relationships between organs, such as muscle-to-muscle and muscle-to-bone, in a biofidelic manner for a body model with multiple muscles.

This work presents the development of an efficient framework for modeling 3D active skeletal muscles at a system level to simulate realistic joint motion (see Supplementary Material for the flowchart of implementation process). The muscles were modeled as active, quasi-incompressible, transversely isotropic and hyperelastic solids, which were implemented into the commercial FE software ABAQUS/Explicit through user-defined material subroutines (VUMAT). A parametric study was conducted to validate the muscle model with ideal 3D geometry at the tissue level, followed by the modeling of the quadriceps muscle group combined with a previously described detailed FE representation of the human knee (Ali et al., 2016; Hume et al., 2018). To demonstrate the effectiveness of the active muscle model in the lower limb, the system level model performance was demonstrated by simulating knee flexion-extension with active quadriceps in a lower limb model. Computational fluid dynamics (CFD) was employed to specify fiber directions for the quadriceps muscles. The interactions between adjacent muscles, and between muscles and femur were innovatively simulated using the cohesive contact approach in ABAQUS. This allows sliding between parts under shearing but restricts separation between the involved parts under tension.

## 2 Methods and materials

### 2.1 Constitutive modeling of skeletal muscle

**FIGURE 1:**
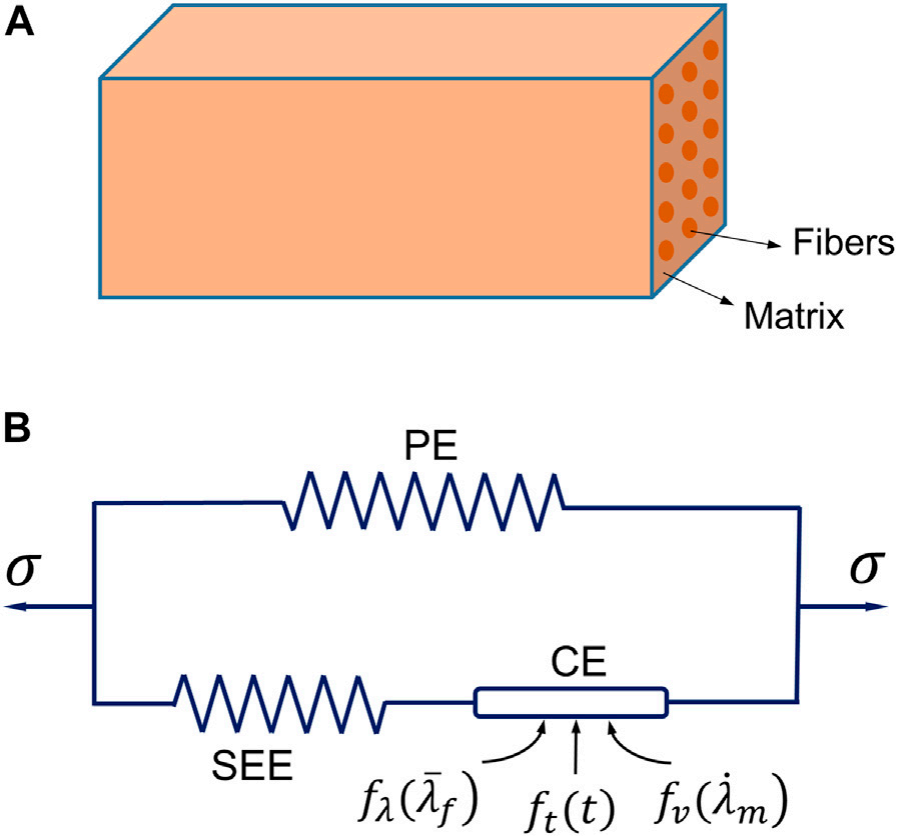
The three-element Hill’s muscle model: **(A)** illustration of the model as a fiber-reinforced composite, and **(B)** the schematic diagram of the model consisting of three elements [a parallel element (PE), a series elastic element (SEE) and a contractile element (CE)].

The constitutive model is the core of the physics of modeling active skeletal muscle. The muscle constitutive law used in this work was based on the strain energy approach, and the general framework of the Hill-type hyperelastic active muscle model was derived from previous studies (Blemker et al., 2005; Tang et al., 2009; Lu et al., 2011a; Khodaei et al., 2013; Kleinbach et al., 2017). The muscle was modeled as a fiber-reinforced composite (Figure 1A) in which the strain energy density function includes the contributions from the composite and the contributions from the fibers (Blemker, 2017). The strain energy density function of active muscle can be expressed by an additive form:

$$W=W_{I}+W_{f}+W_{V}$$ (1)

where $W_{I}$ , $W_{f}$ , and $W_{V}$ represent the strain energy densities for the muscle matrix, the muscle fiber and related volume change, which can be given by the three equations as follows (Tang et al., 2009)

$$W_{I}=c\left\{\exp\left[b\left({\bar{I}}_{1}^{C}-3\right)-1\right]\right\}$$ (2)

in which the ${\bar{I}}_{1}^{C}$ is the first invariant of the right Cauchy-Green strain tensor ( C ), *b* and *c* are material parameters associated with the matrix (Chen and Zeltzer, 1992)

$$W_{f}=\int_{1}^{{\bar{\lambda }}_{f}}\left[\sigma_{PE}\left(\lambda \right)+\sigma_{SEE}\left(\lambda ,\lambda_{s}\right)\right]d\lambda$$ (3)

where $\sigma_{PE}\left(\lambda \right)$ signifies the stress contributed by the parallel element (PE), $\sigma_{SEE}\left(\lambda ,\lambda_{s}\right)$ is the stress contributed by the series elastic element (SEE), *λ* refers to the fiber stretch ratio, ${\bar{\lambda }}_{f}$ represents the fiber stretch ratio with the volume change eliminated, i.e., modified fiber stretch ratio, and $\lambda_{s}$ represents the stretch ratio in the SEE.

$$W_{V}=\frac{1}{D}{\left(J-1\right)}^{2}$$ (4)

where D is the constant of compressibility, and J denotes the Jacobian of the deformation gradient.

The stress σPEt+Δt produced by the parallel element (PE) of the muscle at time t+Δt can be expressed by

σPEt+Δt=σ0fPEλ¯ft+Δt (5)

where $\sigma_{0}$ is the maximum isometric stress, and the function fPEλ¯ft+Δt can be defined by (Chen and Zeltzer, 1992)

fPEλ¯ft+Δt=Aλ¯ft+Δt−12,0,forλ¯ft+Δt>1otherwise (6)

in which A is the parameter related to the stress in the PE.

Accordingly, the stress produced by the SEE at time t+Δt is specified as (Pinto and Fung, 1973; Fung, 1993; Tang et al., 2009)

σSEEt+Δt=βeαλst+Δt−1−1=eαΔλsβeαλst−1−β=eαΔλsσSEEt+β−β (7)

with

σSEEt=βeαλst−1−1 (8)

where α and β are the material constants associated with the stress in the PE, and the relation λst+Δt=λst+Δλs was used in the derivation process for Eq. 7.

The stress produced in the contractile element (CE) is defined as

σCEt+Δt=σ0⋅fλλ¯ft⋅fvλ˙m⋅ftt+Δt (9)

where the fλλ¯ft , $f_{v}\left({\dot{\lambda }}_{m}\right)$ , and $f_{t}\left(t+\Delta t\right)$ denote the function of muscle stretch, the function of muscle velocity, and the muscle activation function, respectively. Their mathematical expressions are given by the equations below (Gordon et al., 1966; Böl and Reese, 2008):

fλλ¯ft=0,9rfλ−0.42,1−41−rfλ2,9rfλ−1.62,0,ifrfλ<0.4if0.4≤rfλ<0.6if0.6≤rfλ<1.4if1.4≤rfλ<1.6ifrfλ≥1.6 (10)

where the dimensionless constant rfλ=λ¯ft/λopt , and $\lambda_{opt}$ is the optimal fiber stretch at which the sarcomeres reach optimal length (Blemker et al., 2005).

It is generally known that the force decreases with increasing velocity during concentric muscle contraction (Hill, 1970; Fung, 1993). The hyperbolic function of muscle velocity $f_{v}\left({\dot{\lambda }}_{m}\right)$ can be expressed in terms of the ratio of stretch rates $r_{m}^{\lambda }={\dot{\lambda }}_{m}/{\dot{\lambda }}_{m}^{\min}$ as (Böl and Reese, 2008)

$$f_{v}\left({\dot{\lambda }}_{m}\right)=\left\{\begin{array}{l} \frac{1-r_{m}^{\lambda }}{1+k_{c}r_{m}^{\lambda }},\\ d-\left(d-1\right)\frac{1+r_{m}^{\lambda }}{1-k_{c}k_{e}r_{m}^{\lambda }}, \end{array}\right.\begin{array}{l} \begin{array}{cc} if & {\dot{\lambda }}_{m}\leq 0 \end{array}\\ \begin{array}{cc} if & {\dot{\lambda }}_{m}>0 \end{array} \end{array}$$ (11)

where ${\dot{\lambda }}_{m}$ is stretch rate in the CE, ${\dot{\lambda }}_{m}^{\min}$ stands for the minimum stretch rate, $k_{c}$ and $k_{e}$ are dimensionless parameters controlling the curvature of the concentric phase (shortening) (Hill, 1938) and eccentric phase (lengthening) of the curve, and d is a dimensionless constant to describe the offset of the eccentric function.

Muscle activation behavior is complicated and further investigation of activation patterns is needed (Ting et al., 2012; Roux et al., 2021). In this work, an exponential function has been employed to characterize muscle activation (Meier and Blickhan, 2000):

$$f_{t}\left(t\right)=\left\{\begin{array}{l} n_{1},\\ n_{1}+\left(n_{2}-n_{1}\right)\cdot h_{t}\left(t,t_{0}\right),\\ n_{1}+\left(n_{2}-n_{1}\right)\cdot h_{t}\left(t_{1},t_{0}\right)-\left[\left(n_{2}-n_{1}\right)\cdot h_{t}\left(t_{1},t_{0}\right)\right]\cdot h_{t}\left(t,t_{1}\right), \end{array}\right.\begin{array}{l} \begin{array}{cc} if & t\leq t_{0} \end{array}\\ \begin{array}{cc} if & t_{0}<t\leq t_{1} \end{array}\\ \begin{array}{cc} if & t>t_{1} \end{array} \end{array}$$ (12)

with

$$h_{t}\left(t_{j},t_{k}\right)=\left\{1-\exp\left[-S\cdot \left(t_{j}-t_{k}\right)\right]\right\}$$ (13)

where $t_{0}$ and $t_{1}$ denote the activation time and the deactivation time of the muscle, separately, $n_{1}$ represents the activation level before and after the activation, $n_{2}$ denotes the activation level during the activation process, and S is the exponential factor (Lu et al., 2010).

Since the stresses produced in the SEE and CE are identical (Figure 1B), the unknown $\Delta \lambda_{s}$ can be obtained by Equations 7 and (9), which leads to the governing equation to solve the stress increment of SEE (Tang et al., 2009):

$$f\left(\Delta \lambda_{s}\right)=\left(w_{2}+w_{3}\Delta \lambda_{s}\right)e^{\alpha \Delta \lambda_{s}}-w_{4}\Delta \lambda_{s}-w_{5}=0$$ (14)

where for muscle shortening (concentric phase)

w2=β+σst1+kc⋅w1λ˙mmin⋅Δt (15)

w3=−β+σstk⋅kcλ˙mmin⋅Δt (16)

w4=−β⋅kc+fλλ¯ft⋅ftt+Δtλ˙mmin⋅Δtk (17)

w5=β+fλλ¯ft⋅ftt+Δt−fλλ¯ft⋅ftt+Δt−β⋅kcλ˙mmin⋅Δtw1 (18)

with

w1=1+kλft+Δt−λmt−ktλs (19)

and for muscle lengthening (eccentric phase)

w2=β+σst1−kc⋅ke⋅α1λ˙mmin⋅Δt (20)

w3=β+σstk⋅kc⋅keλ˙mmin⋅Δt (21)

w4=β⋅kc⋅ke+fλλ¯ft⋅ftt+Δt⋅d⋅kc⋅ke+d−1λ˙mmin⋅Δtk (22)

w5=β+fλtλ¯f⋅ftt+Δt−fλλ¯ft⋅ftt+Δt⋅1−d1+kc⋅ke−β⋅kc⋅keλ˙mmin⋅Δtw1 (23)

where k denotes the ratio of the length of CE to that of SEE, which is set as 0.3 (Lu et al., 2011a).

### 2.2 Parametric study and validation by muscle elongation simulation

The above constitutive model was implemented in user-defined subroutines (VUMAT) using ABAQUS/Explicit, which contains more than 14 material parameters (Table 1). The identification and calibration of these material parameters is a significant aspect of developing the credible muscle model. Based on elongation simulations using both passive and active muscle constitutive models with idealized 3D geometry, the parametric study including investigation of the parameter sensitivity was performed to calibrate and validate the muscle model.

**TABLE 1:** Material parameters used for the muscle model.

Description of parameter		Parameter	Value	References/Source
Compressibility constant		*D* [Pa^−1^]	5.0 × 10^−9^	Parametric study
Stress in matrix		*b*	15	Humphrey and Yin (1987), Martins et al. (2006), and parametric study
		*c* [Pa]	3.79×10^2^	
Stress in PE		*A*	4.0	Chen and Zelter (1992)
		*σ*_0_ [Pa]	7.0×10^5^	Zajac (1989) and parametric study
Stress in SEE		*α*	10	Pinto and Fung (1973)
		*β* [Pa]	1.0×10^3^	
Stress in CE	$f_{\lambda }\left({\bar{\lambda }}_{f}\right)$	$\lambda_{opt}$	1.05	Gordon et al. (1966)
		*k*	0.3	Fung (1993), Lu et al. (2011a)
	$f_{v}\left({\dot{\lambda }}_{m}\right)$	*k*_c_	4	Böl and Reese (2008), Tang et al. (2009), Close (1964), and parametric study
		*k*_e_	5	
		*d*	1.45	
		${\dot{\lambda }}_{m}^{\min}$ [s^-1^]	−1.7×10^1^	
	$f_{t}\left(t\right)$	S [s^-1^]	5.0×10^1^	Meier & Blickhan (2000)

**FIGURE 2:**
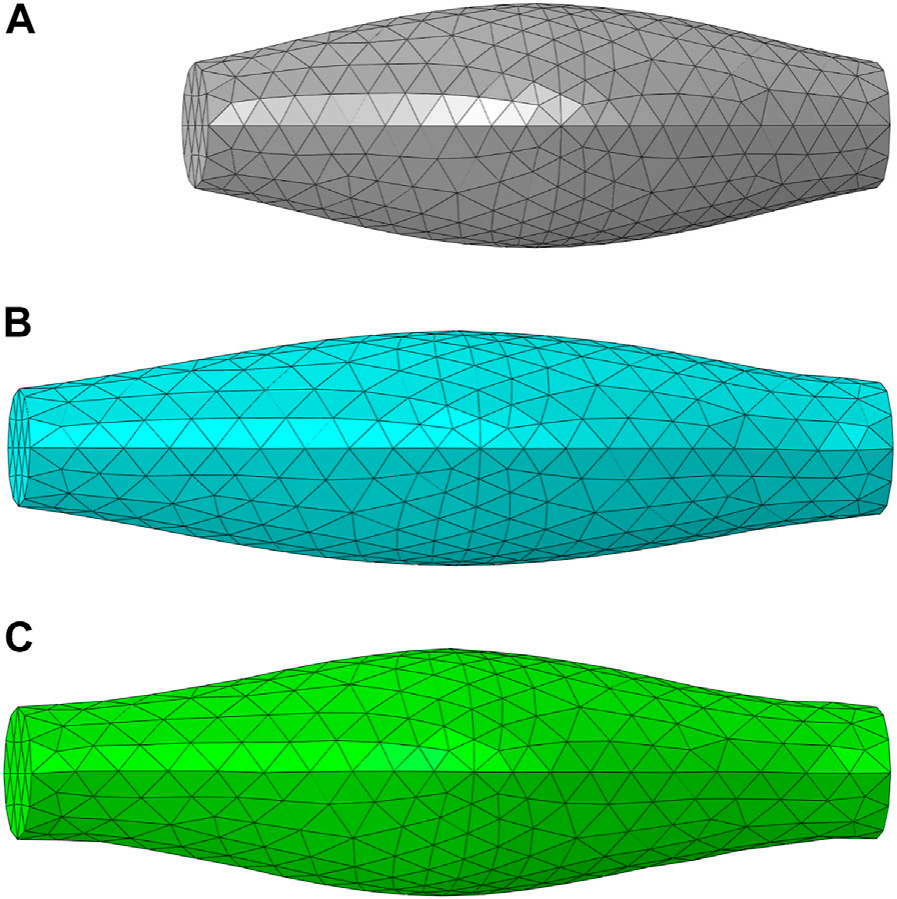
The FE model of muscle with fusiform-shaped geometry: **(A)** initial configuration **(B)** a deformed configuration of passive muscle (20% elongation), and **(C)** a deformed configuration of active muscle (20% elongation) and 100% activation.

The FE model of muscle with fusiform-shaped geometry (Figure 2A), which was adopted in previous studies (Böl and Reese, 2008; Lu et al., 2011a) was created in ABAQUS to simulate the muscle response under uniaxial extension. It has a length of 50 mm, and the diameters of the end section (minimum area) and mid-belly cross-section (maximum area) were set as 9 and 17.5 mm, respectively. The fiber orientation was assumed to be aligned along a uniform direction parallel to the axis of the model, since the fiber dispersion is minor in the fusiform skeletal muscles (Zajac, 1989). The simulations were carried out with two different scenarios: elongation of passive muscle (Figure 2B) and elongation of active muscle (Figure 2C). In the passive simulation, one of the ends of the muscle model was fixed and the other end was pulled with a constant velocity (5 mm/s) for 2 s. In the active simulation, the dimension and mesh were the same as the passive muscle elongation. While the muscle was also fixed on one end, the muscle was held constant in length for 0.5 s to ensure full activation, then the muscle was pulled with a constant velocity of 5 mm/s for 2 s (the muscle was activated during this process). In a similar fashion to the parameter study conducted in one of our authors’ previous work (Lu et al., 2011a), several parameters were determined through parameter studies. These parameters include *D*, *b*, *c*, *d*, $k_{c}$ and $k_{e}$ , as demonstrated in Table 1. The simulated engineering stress response was collected and compared with the available experimental data. In the passive muscle elongation, the stress-strain response was compared with the experimental data reported by Davis et al. (2003), which was measured from the isolated rabbit TA muscle. The active muscle elongation response was compared with the stress-strain response of the active rabbit TA from experiments reported by Myers et al. (1998).

### 2.3 Estimation of the muscle fiber architecture by CFD

**FIGURE 3:**
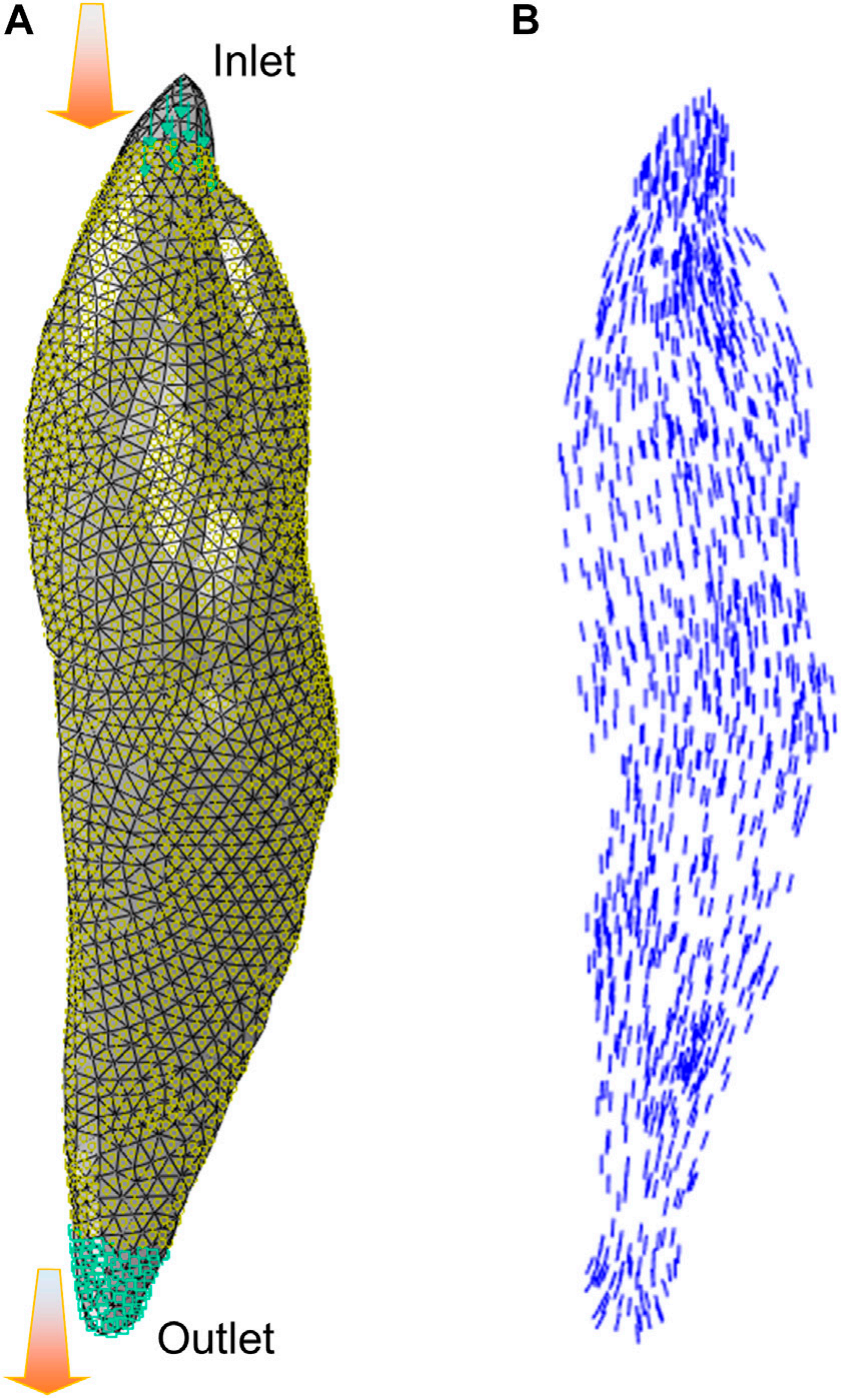
The illustration of CFD fiber simulation: **(A)** flow simulation setup for the rectus femoris (RF) with inlet and outlet boundary condition surfaces, and **(B)** velocity vectors were exported from simulation to characterize the fiber arrangements.

In a CFD simulation aimed at estimating muscle fiber architecture (Inouye et al., 2015), the regions of muscle origin and insertion are designated as the “inlet” and “outlet” of the fluid flow, respectively. Furthermore, the surface of the muscle is assumed to be impenetrable, except for the inlet and outlet, as demonstrated by Handsfield et al. (2017). The resultant velocity vector field produced by the CFD model can then be used to represent muscle fiber architectural arrangements. In some recent studies, the flow vectors predicted from the CFD fiber simulation approach were compared with fiber fields determined by diffusion tensor imaging (DTI), which exhibited similar fiber maps (Varvik et al., 2021). Figure 3 shows an example of CFD fiber simulation for the rectus femoris (RF) using ABAQUS/CFD (CAE Version 6.13–4). In the simulations, the origin and insertion served as inlet and outlet boundary condition surfaces of the Newtonian flow with an inlet velocity. The velocity vector map of the simulated flow was then processed to estimate the map of fiber directions.

### 2.4 Modeling of multiple muscles and anatomical interactions: quadriceps response in knee flexion-extension test

**FIGURE 4:**
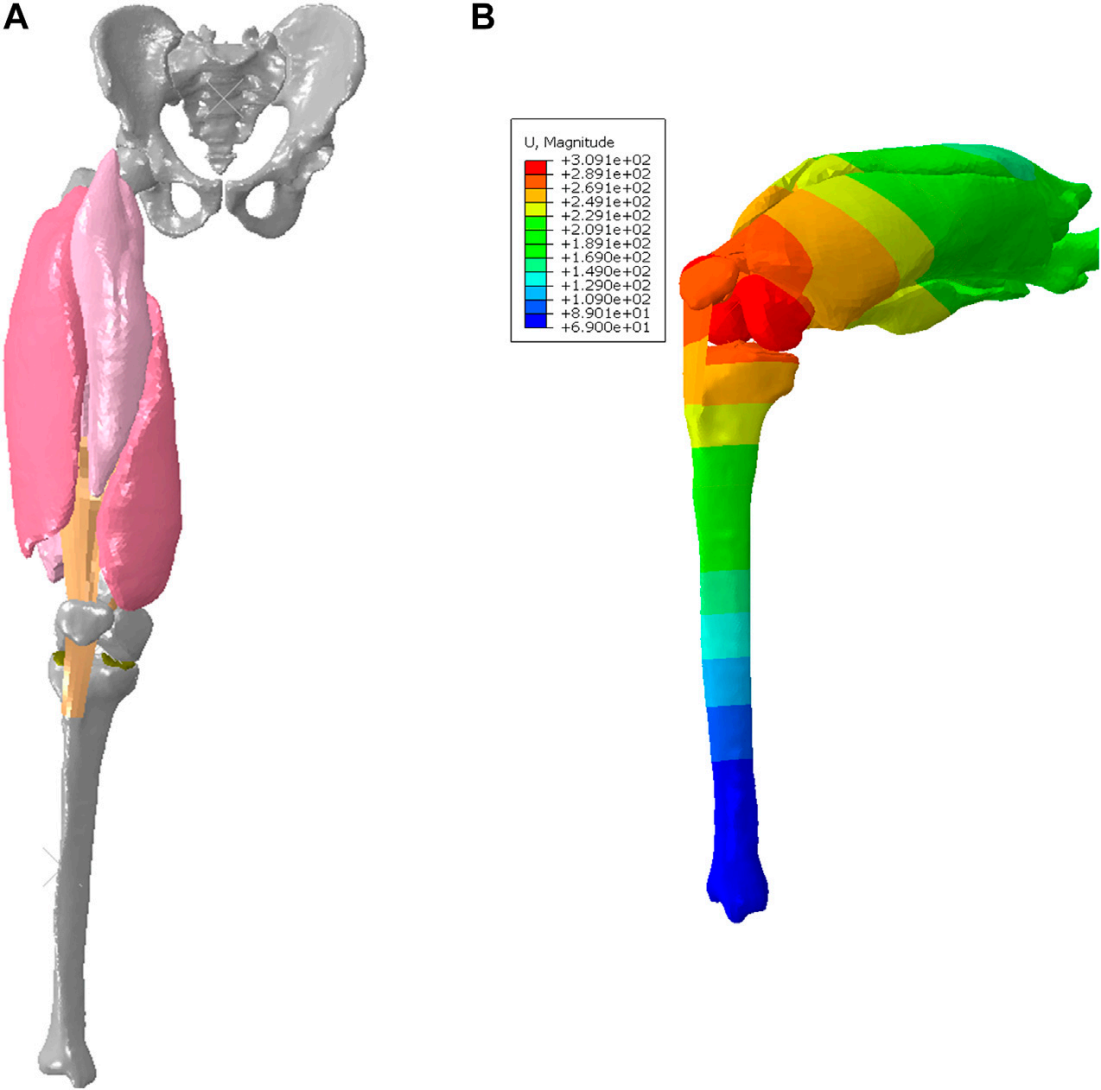
FE model with deformable quadriceps to simulate knee flexion-extension: **(A)** configuration of initial position (full extension), and **(B)** deformed configuration with displacement map for 90 deg knee joint flexion during the squat.

Regarding 3D active skeletal muscle simulation, most current models have been developed with a single muscle configuration (Dao and Tho, 2018). Only a limited number of studies integrated multiple active skeletal muscles into a body region (Stelletta et al., 2017). In this study, we extended the 3D active muscle model presented above into a body system level with multiple lower extremity muscles (Figure 4A), aiming to examine the effectiveness and stability of simulating motion with multiple instances of the above presented pipeline for single active muscle modeling. Simulation of the quadriceps mechanism including the knee is important for the investigation of muscle pathologies and treatments that impact lower limb function, such as acute injury (Puladi et al., 2022), fibrosis (Hung et al., 2014), and sarcoma (Houdek et al., 2021). To simulate active quadriceps, we have a baseline model of the right lower limb which included subject-specific bony structures and cartilages of the knee segmented from computed tomography (CT) and magnetic resonance imaging (MRI) (Hume et al., 2019). It has a 3 DOF ball-joint representing the hip joint, and 6 DOF joints to characterize the patellofemoral (PF) and tibiofemoral (TF) joints (Harris et al., 2016). The patellar tendon and patellofemoral ligaments were modeled as hyperelastic quadrilateral meshes embedded with nonlinear springs (Hume et al., 2019). The 3D deformable active quadriceps muscles were created using material parameters calibrated in the previously presented simplified model, including vastus intermedius VI), vastus lateralis (VL), vastus medialis (VM), and rectus femoris (RF). The 3D geometries of the quadriceps muscles were segmented from images of the Visible Human Male (Ackerman, 1998; Andreassen et al., 2023). The fiber distributions of each individual muscle were modeled using the CFD simulation method described in section 2.3. A knee flexion-extension during a squat was simulated in ABAQUS/Explicit from full extension to 90 deg knee joint flexion, then extended back to full extension. To achieve the flexion-extension motion of the knee in a squat, the pelvis was assigned a kinematic profile in the vertical direction. The distal end of tibia was pinned, and tibial rotation was permitted in the sagittal plane. At approximately 35° of knee flexion, the quadriceps muscles were activated to 50% of their maximum level (Krishnan et al., 2011; O'Bryan et al., 2022) and this level was maintained until maximum flexion was achieved.

It remains a computational challenge to model interactions among muscles. To simulate realistic interactions (Stecco et al., 2008), a computational solution should restrict separations between muscles under tension, but allow relative sliding caused by shearing. A feasible approach is to employ cohesive contact (CC) approach in ABAQUS, which has been used to model bonded interfaces with the possibility of damage or failure in fracture mechanics (Kulkarni et al., 2021). After implementation of the approach using several simplified models, the cohesive contact approach was then utilized to simulate the muscle interactions in the current quadriceps model. The parameters of the cohesive properties used in this study include: the stiffness coefficients *K*_nn_ = *K*_ss_ = *K*_tt_ = 0.05 N/mm^3^, and the damage initiation (maximum separation) *D*_nn_ = *D*_ss_ = *D*_tt_ = 30 mm. The approach can indeed transmit loads normal to the boundary of part, which restricts separation of anatomical structures by tension, but permits relative sliding. In this study, the effective stress (Zeng et al., 2021a) was reported and can be compared with previous studies (Li et al., 2019). In this study, the 95th percentile of von Mises stress (95% vM stress) based on the 95th percentile peak element response was used to characterize the model outcomes (peak stress means 95% vM stress hereafter). Similar to the idea of using 95th percentile maximum principal strain (MPS) in brain tissue biomechanics (Panzer et al., 2012), 95th percentile von Mises stress may avoid or limit potential numerical artifacts on a single element or very few elements associated with using the 100th percentile-ranked maximum element value (Gabler et al., 2019).

## 3 Results

In this section, some key results are provided for both validation of single muscle elongation and response of a group of muscles (quadriceps) in knee flexion-extension.

### 3.1 Passive and active behavior validation by elongation tests

**FIGURE 5:**
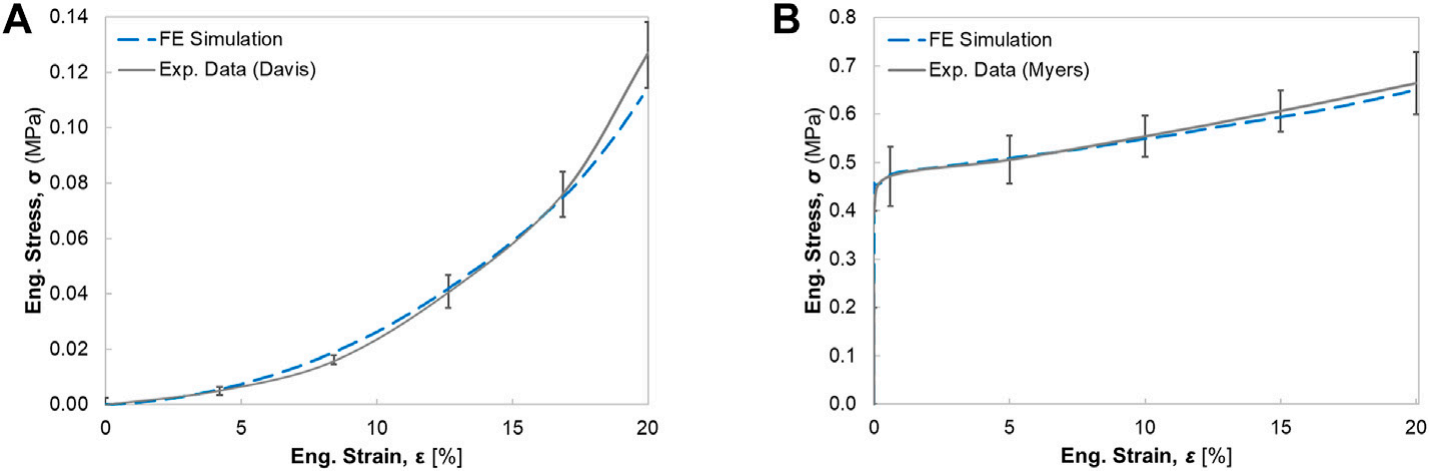
The engineering stress-strain response for the single muscle under uniaxial elongation: **(A)** comparison of passive muscle response with experimental data from Davis et al. (2003) **(B)** comparison of active muscle response with experimental data from Myers et al. (1998).

For uniaxial tensile tests, the deformed configurations (20% elongation) of passive muscle and active muscle were compared with the experimental testing results. Differences of deformation were found between passive and active elongation simulations (Figures 2B,C). The sensitivity analysis showed that the compressibility constant *D* and the maximum isometric stress $\sigma_{0}$ have considerable influence on the engineering stress response, which indicates that it is critical to choose appropriate values for *D* and $\sigma_{0}$ (Lu et al., 2011a). The simulated responses agreed well with the corresponding experimental curves (Myers et al., 1998; Davis et al., 2003), and only slight differences were observed (e.g., the curves with more than 15% engineering strains) (Figure 5).

### 3.2 Response in knee flexion-extension

**FIGURE 6:**
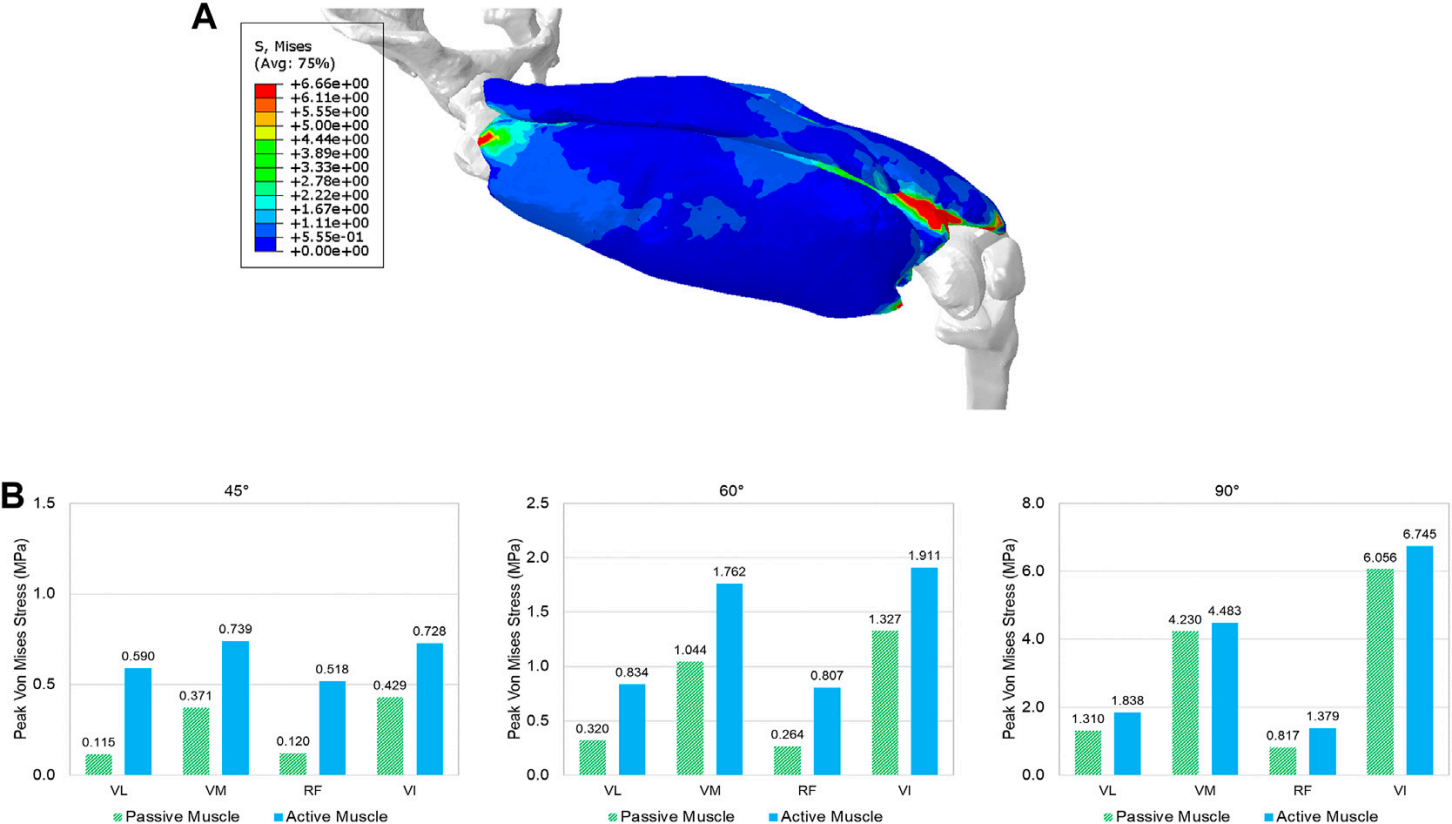
The predicted effective stress in the quadriceps: **(A)** the stress distribution in the quadriceps muscles at the 90° knee joint flexion, and **(B)** comparison of the peak stress values (95% percentile of von Mises stress) between passive and active muscles for different angles (30°, 45°, 60°, and 90°) of knee extension (VL: vastus lateralis, VM: vastus medialis, RF: rectus femoris, and VI: vastus intermedius).

The deformed configuration with displacement map for 90 deg knee joint flexion was displayed in Figure 4B, which can be compared with the initial configuration with 0 deg of knee extension. Figure 6A provided the configuration of the deformable active muscles along with the rigid bones, as well as the stress distribution (up to the peak stress) in the muscles of the quadriceps under the 90 deg knee joint flexion. For the peak stress, it occurred in the ends of muscles (e.g., insertions and origins), as well as some interaction regions between anatomical structures due to contacts (e.g., the contacted areas between distal parts of VI and VM). The comparisons of the peak stress values (i.e., 95% vM stress) between passive and active muscles were collected in Figure 6B. It can be noticed that the peak stress was mostly located at the distal ends of VI and VM, likely due to the large tension in this region and associated muscle-muscle interactions and muscle-bone interactions. It is apparent that the maximum magnitude of the peak stress in each muscle produced by the model with active quadriceps was higher to a certain extent than the peak stress produced by the passive muscle model. Compared to the musculoskeletal FE model of lower extremity developed previously (Li et al., 2019) for kinematics during walking gait, the peak stresses predicted by the models in the current study were lower, but of a similar magnitude (MPa). However, the maximum stresses in Li et al. (2019) were calculated without using 95th percentile maximum element response of the stress, which can potentially be affected by numerical artifacts with using the 100th percentile value (Carlsen, et al., 2021).

## 4 Discussion

This study presented a framework for modeling active muscles by 3D continuum approach and incorporated multiple realistic muscles with interactions into a lower limb that is capable of simulating lower extremity motion. The general computational framework of Hill-type hyperelastic 3D active muscle model was presented, which integrated passive and active properties of muscle into a continuum material model based on a strain energy approach (Blemker et al., 2005; Tang et al., 2009; Lu et al., 2011a; Khodaei et al., 2013; Kleinbach et al., 2017). Compared to discrete approaches to model the muscle using 1D beam/truss elements combined with a 3D solid matrix, the presented framework can deal with complex geometries and is more convenient to implement without the tedious process to define muscle fibers restricted by the mesh of the matrix (Feller et al., 2016; Hedenstierna et al., 2008; Mo et al., 2018 and 2019). To estimate the muscle fiber arrangements, the CFD fiber simulation approach was used and applied for muscles with 3D realistic geometry. The model of muscle was then implemented at a system level into a model of the quadriceps with multiple active muscles and detailed representation of the knee joint under knee bending conditions, which demonstrated the effectiveness and numerical stability of the presented muscle model for simulating complex musculoskeletal systems. The cohesive contact approach was adopted to model interactions between muscles and between muscle and bone. To the authors’ knowledge, this work is the first study which utilized a cohesive contact approach for modeling interactions among anatomical structures in a dynamic simulation with faithful 3D muscle geometry.

Despite the accurate representation of the skeletal muscle geometry and fiber architecture, parameter identification or determination is another challenge of modeling realistic active muscles (Dao and Tho, 2018). The material parameters of different active skeletal muscle models exhibited a wide range of values and variations, likely due to different skeletal muscle specimens used and differences in the experimental methods and modeling approaches. This study describes the active muscle model by multiple constitutive parameters, which characterize the stress in the matrix, PE, SEE, and/or CE. The uniaxial elongation simulations of both passive and active muscles using 3D ideal geometry were conducted to identify the sensitivity of main parameters (also see Lu et al., 2011a) and determine the values of the adjustable material parameters. Compared with the simulation results in Lu et al. (2011a), the presented muscle model in this study with calibrated material parameters displayed a closer correlation with the experimental curves in Figure 5, particularly for large engineering strain (e.g., >10%). Regarding the definition of fiber orientations, the physics-based approach using CFD simulation was utilized to model the subject-specific muscle fiber arrangements. The method was compared against the fiber orientations observed with DTI (Inouye et al., 2015; Handsfield et al., 2017; Varvik et al., 2021), which has shown to be a physiologically reasonable and cost-effective simulation approach for predicting fiber trajectories. Compared to the other modeling approaches to create the fiber map such as mapping technique from different fiber templates (Blemker et al., 2005), the CFD fiber simulation technique is computationally more efficient and can handle subject-specific and muscle-specific differences without template-confined limitations such as the requirement of a structured mesh.

Few simulations have been reported using active 3D skeletal muscle models, and most of the loading cases used simplified loading conditions, such as single muscle elongation, shortening, or contraction (Dao and Tho, 2018). In the context of modeling multiple active muscles behaviors, it is still comparatively rare and almost all prior methods adopted 1D discrete elements to model the active properties (Feller et al., 2016; Mo et al., 2018) with a combination of continuum elements for passive properties (Khodaei, et al., 2013). If the active muscle model is only simulated with very simple geometry under basic loading scenarios (e.g., uniaxial tension, shearing, shortening, or lengthening), the numerical stability and effectiveness of the model might be overlooked for multiple realistic muscles with large deformation within a body region or even a whole human body model. In this study, the presented method for single active muscle modeling has been extended to the quadriceps under dynamic simulation of knee bending at a system level, which involved multiple active muscles and interactions among muscles. Modeling and simulation of interactions between anatomical structures (e.g., muscles) is still challenging in several ways, including mechanical and physiological aspects, as well as numerical issues. While a small number of prior studies have included multiple 3D muscles, they were limited by either not including contact between muscles or representing contact in non-physiological ways (Stelletta et al., 2017). For example, the quadriceps were modeled without considering contacts or other interactions between muscles (Fernandez and Hunter, 2005). The common approaches to model interactions between anatomical structures include sharing nodes between the mesh of structures (e.g., the Global Human Body Models Consortium (GHBMC) extremity models (Schwartz et al., 2015; Zeng et al., 2021b), fixed constraint(s) between organs (Fernandez et al., 2005), contact (Stelletta et al., 2017), and combination of contact and springs (Böl et al., 2011). Physiologically, the muscles of the lower extremity such as quadriceps are enveloped by their deep fascias, permitting relative sliding of the muscles because of their epimysium (Stecco et al., 2008). In this work, for the first time at the quadriceps, we modeled the interactions among anatomical structures using a cohesive contact approach. It can restrict separation of the structures (e.g., muscle to muscle, muscle to bone) under tension, but still permit relative sliding due to shearing. The four bundles of the quadriceps experienced large deformation during the knee bending process (Figure 6A). It should be emphasized that the specific aim of this example was to show the numerical stability and applicability of the presented 3D active skeletal muscle modeling framework for multiple muscle configurations with anatomical interactions at a system level. Because the responses of multiple muscles in a complex system can be affected by a wide variety of factors (e.g., geometry, locations of origins and insertions, attached tendons and ligaments, surrounding structures, interactions, boundary and loading conditions, *etc.*), the current study did not seek a definitive quantification of the stress or strain of the muscles. However, the trends of simulated results agreed with some published studies including a previously developed musculoskeletal FE model of the lower extremity (Li et al., 2019), which showed the stresses were concentrated at the muscle’s insertion and origin regions most likely because of the small muscle cross-sectional area of the two ends of the muscles. Compared to the model presented by Li et al. (2019) for kinematics during walking gait, the peak stresses predicted by the model in the current study were lower, but of a similar magnitude (MPa). However, the maximum stresses in Li et al. (2019) were calculated without using 95th percentile maximum element response of the stress, which can potentially be affected by numerical artifacts with using the 100th percentile value (Carlsen, et al., 2021). The current study also found that large stress levels appeared at the contacted regions between muscles, including the insertion ends of the VI and VM. Additionally, as expected, the peak stress in each muscle with active properties appeared to be higher than the corresponding muscle with passive properties only (Figure 6B). The stress increased in the localized regions of the active muscle since it was stiffened under the active state due to the new contributor of active component integrated with the passive stiffness (Winters, 2012).

Although the present work has yielded promising results, there are some limitations in this study, which can be considered and improved in future research. First, the presented model was validated by the testing data of only two simple loading cases with elongation from rabbit leg muscles, which were the most used muscle type in the literature for model validation and parametric identification. There was a paucity of available experimental data regarding active muscle model validations under different external mechanical stimuli, particularly experimental data (i.e., electrophysiological recordings) of human active skeletal muscles in pathophysiological conditions and for specific aspects of the numerical implementation of the model. Likewise, there is no model validation data developed or presented in the literature for the purposes of the active quadriceps response in knee bending. Additionally, it is possible that the knee angle-dependent differences could be influenced by variations in muscle activation levels (Kooistra et al., 2005). The current study employed the same activation pattern for all muscles, and did not account for the gradient recruitment of fibers along the muscle due to the presence of different neuromotor units within the muscle. Second, there were some model simplifications due to factors such as insufficient representation of anatomical details or material properties. For example, the patellar tendon and patellofemoral ligaments were not calibrated, although the knee joint in the adopted baseline model was validated by comparing kinematics of the FE model with experimental kinematic data (Baldwin et al., 2009; Ali et al., 2016). Third, regarding the characterization of multiple muscle interaction, the quantification of force transmission between muscles remains a challenge from both experimental and simulation perspectives. Although we believe that the presented cohesive contact approach is more efficient and realistic than other existing approaches (Fernandez and Hunter, 2005; Böl et al., 2011; Schwartz et al., 2015; Zeng et al., 2021b) to model organ interactions based on the performance compared to the limited available evidence, the interaction effects on the muscle forces have not been quantified since there is a lack of experimental testing data for validation thus far. Finally, the external fascia and skin were not considered in the current quadriceps model, so further research is needed to accurately represent the thigh to consider the muscle packing effect (Stecco et al., 2008).

## 5 Conclusion

This study proposed a computational framework of 3D muscle simulation by Hill-type hyperelastic active muscle model, which was validated by single muscle loading cases and extended for modeling multiple muscles with interactions during movement at a system level. The presented constitutive model and relevant material parameters were validated by the experimental data based on the rabbit TA muscle (Myers et al., 1998; Davis et al., 2003). The simulation results were in good agreement with the experimental measured elongation responses, which indicated that the current model with determined parameters can characterize the passive and active behaviors of muscles. After conducting single muscle level validation, the active muscle model was implemented into multiple muscle configurations, i.e., the quadriceps response in knee flexion-extension. For each muscle with complex shape, the muscle fiber orientations were defined using the CFD fiber simulation approach. For the interactions among anatomical structures, the effect of contact between muscles and between muscle and femur was qualitatively simulated by the cohesive contact approach, which can restrict separation of the components under tension, but still permit relative sliding due to shearing. The muscle model was stable and effective for modeling multiple realistic 3D muscles with interactions, and capable of predicting the trends of muscle responses through dynamics simulation of knee flexion and extension. Therefore, it is feasible for the presented framework to link the tissue-level active muscle behavior and dynamics of human movements. It is expected that this study can encourage additional computational and experimental studies of skeletal muscle mechanics, particularly muscle activation, quantification of muscle interactions, constitutive model development, parameters determination, and multiscale modeling for neuromuscular dynamic simulation. In a larger scope, this study provides a promising framework toward developing realistic multiscale active human body models for orthopedic treatment, rehabilitation, implant mechanics, and impact biomechanics.

## Data Availability

The original contributions presented in the study are included in the article/Supplementary Material, further inquiries can be directed to the corresponding authors.
