# Supplementary material for: Modeling of active skeletal muscles: a 3D continuum approach incorporating multiple muscle interactions
Source: Front Bioeng Biotechnol. 2023 May 18;11:1153692. doi: 10.3389/fbioe.2023.1153692 (PMC10234509; doi:10.3389/fbioe.2023.1153692)
Supplement: Supplementary file 1 [file DataSheet1.DOCX]

**Supplementary Materials**

**SM-A: Overview of the steps for implementing the current study.**

| **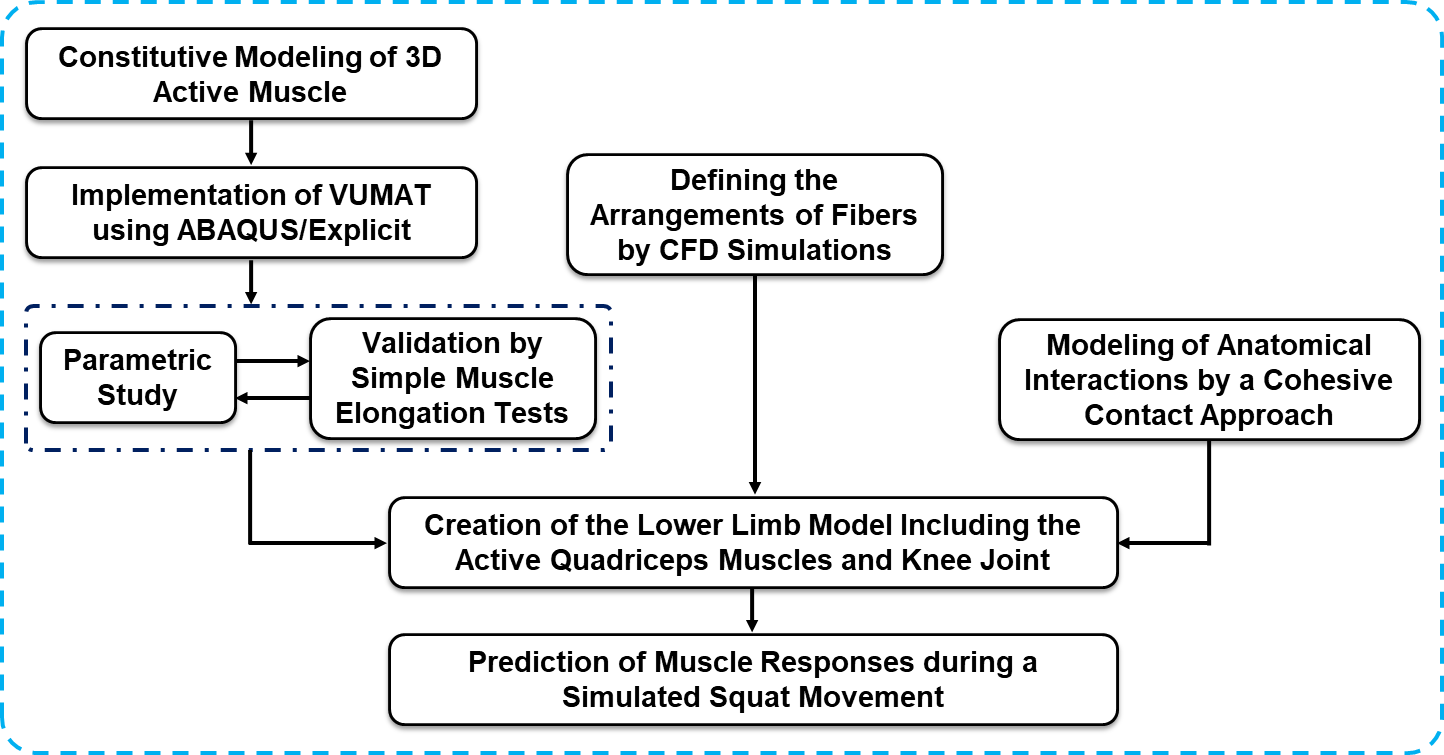** |
| --- |
| Figure SM-1. An overview of 3D active muscle modeling and the inclusion of multiple muscles at a system level to simulate realistic joint motion |
